# Telemedicine: Benefits for Cardiovascular Patients in the COVID-19 Era

**DOI:** 10.3389/fcvm.2022.868635

**Published:** 2022-07-20

**Authors:** Liviu-Nicolae Ghilencea, Maria-Roxana Chiru, Miroslava Stolcova, Gabriel Spiridon, Laura-Maria Manea, Ana-Maria Alexandra Stănescu, Awais Bokhari, Ismail Dogu Kilic, Gioel Gabriel Secco, Nicolas Foin, Carlo Di Mario

**Affiliations:** ^1^Department of Cardiology, Elias University Hospital, Carol Davila University of Medicine and Pharmacy, Bucharest, Romania; ^2^Bedford Hospital NHS Foundation Trust, Bedford, United Kingdom; ^3^Structural Interventional Cardiology, University Hospital Careggi, Florence, Italy; ^4^Department and European Project Development, Institute of Scientific Research and Technological Development in Automation and Informatics, Bucharest, Romania; ^5^Department of Family Medicine, Carol Davila University of Medicine and Pharmacy, Bucharest, Romania; ^6^Department of Cardiology, Bedford Hospital NHS Foundation Trust, Bedford, United Kingdom; ^7^Department of Cardiology, Pamukkale University Hospital, Denizli, Turkey; ^8^Department of Interventional Cardiology and Structural Heart Disease, SS. Antonio e Biagioe Cesare Arrigo Hospital, Alessandria, Italy; ^9^Duke-NUS Medical School, National Heart Research Institute, Singapore, Singapore; ^10^Royal Brompton Hospital, NHSFT, London, United Kingdom; ^11^Department of Cardiology, University of Florence, Florence, Italy

**Keywords:** telemedicine, telemonitoring, COVID-19, cardiovascular disease, implantable devices, heart failure, systemic hypertension and arrhythmia, SWOT analysis

## Abstract

The recent pandemic with SARS-CoV-2 raises questions worldwide regarding telemedicine for housebound patients, including those with cardiovascular conditions. The need for further investigation, monitoring and therapeutic management are advancing practical issues which had not been identified for consideration prior to the pandemic. Using the marketing assessment, we identified the needs of the patients and evaluated the future steps necessary in the short term to meet them. The research found progress made via telemedicine in monitoring and conducting minor decisions (like up-titrating the doses of different medication regimens) in patients with several cardiovascular diseases (heart failure, atrial fibrillation, high blood pressure), as there is a worldwide trend to develop new telemonitoring biosensors and devices based on implantable delivered transcatheter. The worldwide telemedicine trend encourages a switch from small and hesitating steps to a more consistent assessment of the patients, based on high technology and Interventional Cardiology. Cardiovascular telemedicine, although made a sustainable effort in managing patients' health, has many obstacles to overcome before meeting all their needs. Data security, confidentiality and reimbursement are the top priorities in developing remote Cardiology. The regulatory institutions need to play an integrative role in leading the way for defining the framework of future telemedicine activities. The SARS-CoV-2 outbreak with all its tragedy served to reinforce the message that telemedicine services can be life-saving for cardiovascular patients. Once the Covid-19 era will fade away, telemedicine is likely to remain a complementary service of standard care. There is still room to improve the remote identification and investigation of heart disease, provide an accurate diagnosis and therapeutic regimen, and update regulations and guidelines to the new realities of technological progress in the field.

## Introduction

On 25th July 1665, the first casualty of the plague saw the academic community of Cambridge go into lockdown, a similar scenario to that we are facing today. Sir Isaac Newton, then a young student at the University of Cambridge, went into lockdown to his mother's house in Woolsthorpe, Lancashire, due to the great plague outbreak. While he was in the countryside, he had time to think about his ground-breaking ideas regarding the force of attraction and the laws of motion and gravitation. Some 22 years later, Newton's brilliant theories were published in the *Philosophiae Naturalis Principia Mathematica* (Mathematical Principles of Natural Philosophy), laying the foundation of what we know today as Classical Mechanics.

The recent pandemic with SARS-CoV-2 raises questions worldwide regarding telemedicine for housebound patients, mainly cardiovascular disease patients. The need for monitoring, further investigation and therapeutic management are growing practical issues that had not been identified for consideration before the pandemic.

Patients with a known cardiovascular disease or displaying new symptoms or signs of onset (chest pain, shortness of breath, palpitations and peripheral oedema) and those in self-isolation or quarantine raise a public health problem needing a different management approach. In a recent research, during the COVID-19-related lockdown in Germany, a substantial rise in cardiovascular mortality was found (1).

From the clinical perspective, SARS-CoV-2 is increasingly and rapidly affecting Cardiology health care workers, as exposed physicians have to be sent home to self-isolate. Telecardiology could allow affected employees to continue their work from home by providing treatment via virtual meetings and maintaining remote contact with patients and colleagues by telephone. Making a parallel with the classical mechanical situation at the end of the XVIIth century, the SARS-CoV-2 pandemic represents a turning point in telemedicine in which an enlightened mind will bring together the ethical, legal, reimbursement and technical elements, laying the groundwork for what our followers will know as Modern Telemedicine.

## The Needs of Remote Care

Telemedicine, and by extension, telecardiology, consists in providing consultation over a distance using telecommunication strategies to improve the patient's health status (2). Telecardiology employs modern technology for real-time remote diagnosis and treatment of heart disease. It fulfills the needs of the patients for fast, reliable, sustainable and less expensive services. It makes care more accessible, thus increasing healthcare overall and improving outcomes.

Telemedicine is based on the exchange of digital, social and health information to support and optimize the remote care process. The benefit of telemedicine during a pandemic is that the patient can safely stay at home (2). From virtual visits and hospital connections to ongoing monitoring of the patients, the typical mobile health service architecture uses the internet and web services to provide transparent two-way interaction among doctors and patients (2).

Patients who live away from medical facilities, those with mobility difficulties or those who have a busy schedule, as well as those who require continuous medical care and monitoring, are the ones who have the greatest needs for remote cardiology. The patients and medical staff, including physicians, and GPs, may benefit in their decision-making process from highly specialized telecardiology services.

Currently, the patients benefitting the most from the telemedicine approach are those in the following groups:

- Heart failure: monitoring patients' curves of daily body weight, blood pressure, O_2_ saturation, heart rate/rhythm and new invasive direct or indirect assessment of left atrial pressure as a turning point for therapy adjustment.
- Systemic hypertension: diagnosis, monitoring and therapy adjustment via ambulatory blood pressure monitoring.
- Arrhythmia: diagnosis, ECG monitoring and treatment changes including emergency calls in life-threatening situations such as ventricular tachycardia or debilitating conditions like atrial fibrillation.

Since there is not an ideal profile of the patient in need for telemedicine, any cardiovascular patients with systemic hypertension, occult arrhythmia, heart failure or STEMI, especially the fragile or those in remote areas, may be in the position to benefit from the service, whenever standard care proves its' limits. The unfortunate SARS-CoV2 pandemic acted as an opportunity that reinforced and boosted the use of telemedicine in medical practice, not only during lockdown, but also under normal circumstances. To become successful and meet the needs of the patients, any action plan should be based on measures of the contingency plan.

## Telemedicine Groups Definition

We consider telemedicine a heterogeneous area, with several branches at different stages of development and implementation in day-to-day life (Table 1). At the center of remote Cardiology, the patient is the final beneficiary of the medical services, regardless of whether the service is delivered directly or indirectly via medical staff.

**Table 1:** Telemedicine groups and description.

	**Group**	**Example**	**Description**
Real-time online assessment	Virtual video visits of patients (Teleconsultation)	Virtual outpatient's clinic	Patient at home directly connected via online video link with the cardiologist in the hospital
	Second opinion offered by cardiologists to other health professionals (Teleexpertise)	Ambulance crew for patients with chest pain and dynamic ECG changes	Cardiologist on-call confirming the presumption of STEMI and indication to emergency catheterization
		GPs in medical unit/ surgery requesting medical advice for acute patients	In-hospital on-call cardiologists providing skilled support for undiagnosed and inconclusive cases
		General medicine physicians in small hospitals seeking medical expertise	Performing clinical examination via video link and echocardiographic assessment to inpatients
		Online video conference MDT meetings with clinical and interventional cardiologists, cardiothoracic surgeons, radiologists.	Experts of different specialities assessing difficult cases and taking management decisions
Remote monitoring	Wearable devices and implantable biosensors (Telemonitoring)	Heart failure monitoring	Weight, HR, LAP
		Palpitations /syncope monitoring	ECG recording/ events record/ ILR
		Systemic hypertension	BP curve

*ECG, electrocardiogram; MDT, multidisciplinary team; HR, heart rate; LAP, left atrial pressure; BP, blood pressure; GP, general practitioner; ILR, implantable loop recorder*.

According to previous studies, telemedicine refers to remote emergency diagnosis, virtual visits, teleexpertise, and remote cardiovascular monitoring (telemonitoring) (2–13).

***Remote emergency diagnosis*** (***Emergency telediagnosis***) of patients with suspected life-threatening cardiac events, evaluated initially by general practitioners (GPs) or ambulance crew, is defined as a rapid Cardiology assessment service. This is directed toward patients complaining of acute symptoms such as chest pain, palpitations, syncope and dyspnoea and may avoid unnecessary admissions until the hospital setting (4, 5).

Emergency telediagnosis, transmitting the ECG with direct voice contact between ambulance and coordinating center, was implemented more than 20 years ago in many primary angioplasty networks. In the Western Denmark network, it is found to increase the confidence of the mobile ambulance crew to bypass a nearby A&E to drive the patient maybe for an hour to the primary center. The central allocation medical operator could also warn the emergency catheterization laboratory that a new STEMI patient was coming, specifying the territory of interest, hemodynamic status and possible comorbidities so that everything was ready in place by the time he arrived (6, 7). A prehospital 12-lead electrocardiogram reduces the door-to-needle time, with early catheterization lab activation (8, 9).

Furthermore, in the United Kingdom, university hospitals are already providing remote cardiology expertise to GPs in the surrounding areas. This support is delivered via email (formerly by facsimile), with an on-call cardiologist or designated trained cardiologist carefully analyzing the cases (including the ECG) and responding to primary care for a decision-making process (10, 11).

***Remote consultation (or Teleconsultation)*** relates to the virtual visit of a patient with heart disease as it is performed directly by a trained cardiologist. It replaces the physical visit of the patient to the outpatient clinic (OPC) and consists of a virtual video visit (telehealth) directed toward elective patients, with or without remote transmission of vital signs. The patient is logged in to the virtual outpatient clinic, face-to-face in real-time with the cardiologist.

Teleconsultation was initially a surrogate of specialized consultative care in rural areas where doctors' shortages made it difficult for heart patients to receive timely care. Teleconsultations are especially beneficial for those patients who live far from a cardiology office, offering protection against potential infection with SARS-CoV-2 and saving time as well as money.

***Remote expertise (or Teleexpertise)*** in support of primary care for complex cardiovascular issues provides a second opinion via real-time video consultation with the cardiologist at one end and the general practitioner (and the patient) at the other end of the line.

One sub-type of teleexpertise can be the virtual video multidisciplinary team (MDT) meeting, a setting where general physicians or clinical cardiologists can connect remotely, in real-time with physicians of different specialties, including radiologists and cardiothoracic surgeons. The benefit is a range of expert opinions working together to deliver accurate diagnoses and formulate a treatment plan for complex heart conditions following appropriate patient referral, thus avoiding unnecessary movements of patients or medical staff.

***Telemonitoring (or remote cardiovascular monitoring)*** addresses patients at risk who are known to have chronic conditions (heart failure, systemic hypertension and arrhythmias). It is the most rapidly developing area of telecardiology and is based on wearable or implanted biosensors/devices which collect data such as cardiovascular parameters (body weight, blood pressure, heart rhythm, heart rate, glycaemia, oxygen saturation and left atrial pressure) from the patients. The devices worn by patients transmit alerts or alarms directly to the cardiologist (2).

The telemonitoring screening systems are both opportunistic and systematic. The opportunistic wearable devices, like smart-watches, smartphones, pedometers, have boosted the individual telemonitoring, especially under 65 years of age, but few applications are validated in medical care. On the other hand, the systematic devices, both wearable and implantable, like: the 3-6-12-lead ECG, the Implantable Loop Recorder (ILR), the Vectorious-Left Atrial Pressure (V-LAP), and the implantable pulmonary artery pressure device (CardioMEMS), can be used for both screening/diagnostic and management purposes.

From the first electrocardiogram transmitted by phone in 1906 by Einthoven–a moment that marks the birth of telemedicine, and the first timid steps in monitoring the vital functions of the first astronauts in the 60s, telemonitoring has never seen such a development as in the last 25 years (12). The commercial development of wearable devices together with the advances of implantable devices has moved the frontier of knowledge into the core of telemedicine for the benefit of the patients.

Remote cardiovascular monitoring started more than 20 years ago (in the late 1990s) by monitoring heart failure patients' vital signs (heart rate, blood pressure and body weight) and alerts sent to their cardiologist.

Since then, also before the SARS-CoV-2 pandemic, telemonitoring has taken tremendous steps forward, with implantable biosensors added to wearable devices for a more accurate and new parameters assessment. Epigastric subcutaneous implantable loop recorder (ILR) has been used for more than a decade to identify the cause of syncope for patients with seldom events. The use of an implantable pulmonary artery pressure device (CardioMEMS) to provide daily pulmonary artery hemodynamic information showed significant reduction of rehospitalization in NYHA class III heart failure patients in randomized trials (13).

The Vectorious-Left Atrial Pressure (V-LAP) wireless sensor, a second-generation heart failure monitoring system implanted in the interatrial septum, showed promising results in monitoring left atrial pressure. This demonstrates a good correlation with pulmonary capillary wedge pressure (PCWP) in both chronic and acute assessments, and during acute saline loading, in an animal study (14).

## SWOT Analysis

**Figure 1:**
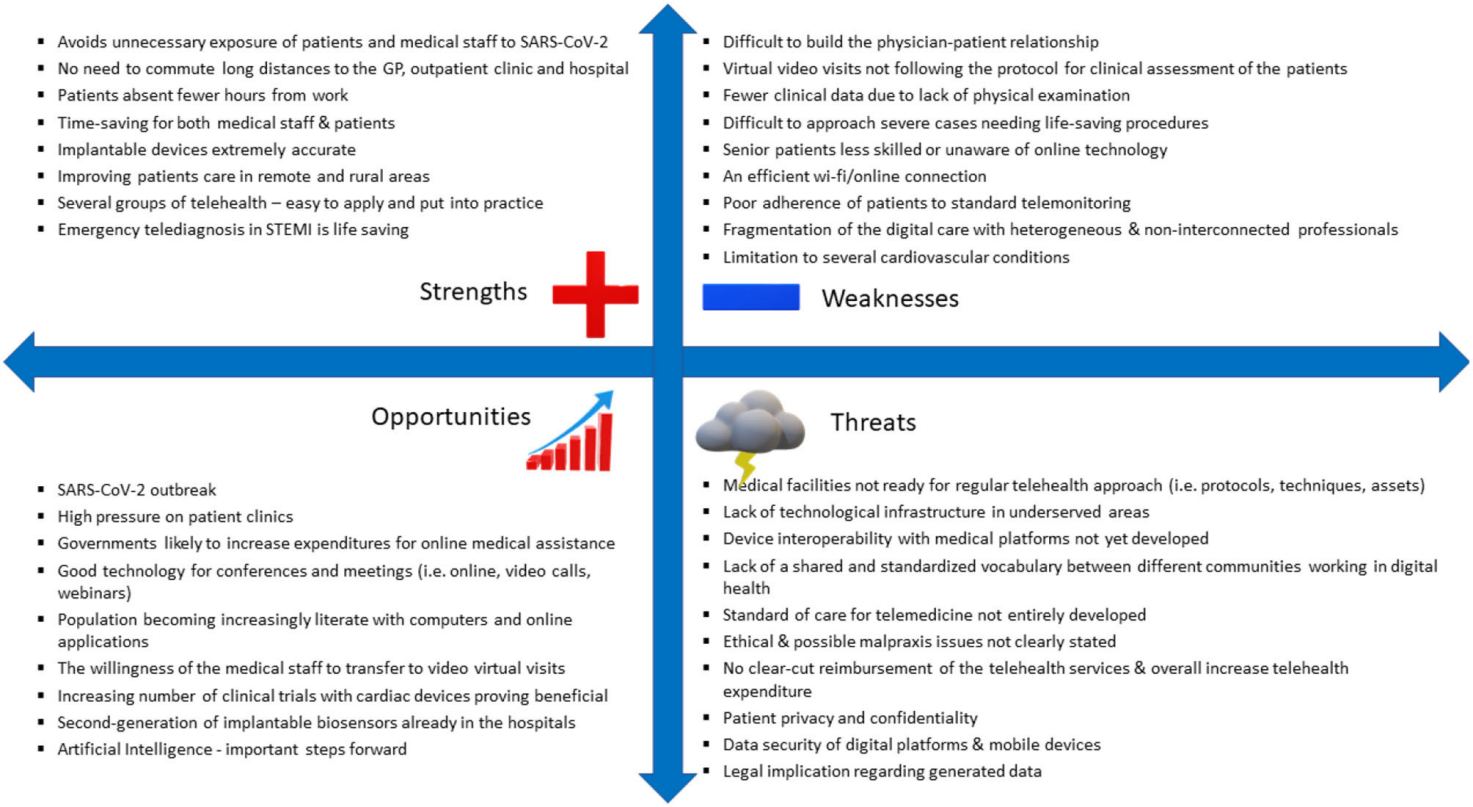
The strengths, weaknesses, opportunities and threats (SWOT analysis) of telemedicine (1–11, 13–17).

The SWOT marketing analysis performed for telemedicine services refers to its' strengths, weaknesses, opportunities, and threats in meeting the needs of the patients. It describes the key issues of the market of medical services (Opportunities and Threats) and the critical success factors (Strengths and Weaknesses) of telemedicine, as is presented in the below example (see Figure 1). The current barriers of telemedicine are presented as Weaknesses and Threats.

Initially developed to increase access to cardiovascular care for patients in remote and underserved areas, telemedicine can provide a good service to patients in quarantine and self-isolation during times such as the ones we are currently experiencing. At one end of the line, there will always be a skilled and trained cardiologist, and at the other, it could be an emergency worker, GP, nurse or the patient himself.

Nowadays, the virtual video visits limit the exposure of the health professional to SARS-CoV-2, replacing the face-to-face meetings in the outpatient clinic with a virtual video visit. Moreover, the virtual video meetings between the cardiologist and the patient can include the local GP as a link to the examination of the patient. Thus, GPs may connect with specialists in real-time to diagnose and treat heart conditions for in-patients in a first-line COVID-19 hospital.

Furthermore, MDT meetings with cardiothoracic surgeons and interventional cardiologists are already held online virtually, thus negating the need for surgeons to commute to hospitals without surgical facilities and specialists. For patients who live hours from a cardiologist, teleconsultations for initial and ongoing visits offer time and money-saving benefits. Such consultations also dramatically reduce both the risk of contracting SARS-CoV-2 and the high pressure on OPC.

The patients hospitalized in local hospitals benefit of the input in diagnosis and therapy from an off-site clinical interventional cardiologist. The patients are monitored remotely through electronic monitoring equipment which prescribes and advises appropriate intervention for the on-site doctor.

For the time being, the possible shortcomings and limitations seem numerous and more related to the ethical, legal and financial aspects. There is to note that repeated hospitalizations are the main drive of the enormous costs (17). Although there is an opportunity to accelerate the development of remote Cardiology, there are many environmental threats that act as potential obstacles. These are likely to delay its application and implementation in the real world, thus slowing down its potential to save lives. It is obvious from the SWOT analysis that most of the threats refer to legal, data safety and ethical issues, while the opportunities are related more to the progress of the techniques used. One of the key issues in SWOT analysis is reimbursement, subject to the decision of regulatory bodies to accept telemedicine as a medical service.

The contingency plan should include appropriate measures to mitigate the Weaknesses and Threats, necessary to be adopted when using telemedicine services, *inter alia*:

- Strict compliance with the related medical standards (DICOM, HL7, ISO / IEEE 11073);
- The use of the appropriate telemedicine equipment that meets medical standards in the field, only;
- Ensuring the security and privacy of medical data;
- Ensuring an adequate education and training of the medical staff involved in the use of the equipment, in the processing, interpretation and storage of the medical data;
- Introducing telehealth accreditation for health professionals (18);
- Redesigning clinical models of care and establishing systems to manage telemedicine services on a routine basis (18);
- Providing funding to adequately cover the cost of telehealth (18);
- Supporting all stakeholders with an effective communication and changing management strategy (18).

## Technical Requirements for a Universal Approach to the Telemedicine

The technologies as some natural extensions of our fingers, eyes and ears are successful in providing more healthcare when they fulfill the needs of the patients and the medical staff.

### Conformity With Medical Standards

The impact of the system will be reflected in direct benefits to the patients and the medical care system. It will be demonstrated through clinical trials. The system has to be developed according to international medical standards: (1) Health Level Seven (HL7) Clinical Document Architecture for storing the patient's medical data, (2) HL7 communication protocol to interact with the hospital information, (3) ISO/IEEE 11073 Point-of-care Medical Device Communication to receive data from the Vital Signs monitor devices, (4) Digital Imaging and Communications in Medicine (DICOM) (19–21); it will facilitate the interconnection with other medical information systems in operation, thus assuring a high acceptance and repeatability degree.

### Security and Privacy of Medical Data

A crucial aspect in handling medical data is protecting the security and privacy of these data. Patient identification and medical records cannot be disclosed indiscriminately. Furthermore, different healthcare providers have additional access rights to these medical patient records (MPR). Privacy issues are at concern as telehealth application developers are not providing enough information regarding the way personal data are managed (15). The system must provide necessary mechanisms to protect health information in all requested forms. Data Privacy is ruled by the European Union Directive 95/46/EC (19). The patient has to be asked to authorize the transmission of his/her personal and medical data. Specific measures will be taken (encryption, authentication) to assure medical data security and privacy.

There are also issues to be considered and developed in a video virtual visit: scheduling system to list virtual phone consultation/visits, encountering and documenting the visit, e-prescribing and placing orders (22).

Future steps for short and medium-term assessment are technical, ethical, financial and regulatory-based. Technological development is based not just on new tools and devices for virtual video visits and telemonitoring but also on artificial intelligence for providing new algorithms for self-diagnosis. An important direction in telemonitoring is focusing on the automated decision support systems for predictive prognostic purposes and to take action by giving indications of management (15).

## Telemedicine Existing Data

A multitude of telemedicine randomized, multicentre, controlled clinical trials and prospective controlled trials, as well as observational clinical studies have been developed in the last 10–15 years (Tables 2–4). Heart failure, atrial fibrillation, ventricular tachycardia, and systemic hypertension are the most monitored conditions.

**Table 2:** Summary of different heart failure telemedicine studies.

**Study**	**Design**	**Assessed device/technique**	**Number of patients**	**End-points**	**Results**
HFHC Soran et al. (23)	Randomized, controlled trial Multicentre	Alere Day Link HF Monitoring System (non-invasive device)	315	Treatment failure (rehospitalizations + cardiovascular deaths at 6 months)	No significant statistical differences
Kulshreshtha et al. (24)	Randomized, controlled trial	Remote monitoring equipment (symptoms, weight)	150	Primary: All-cause rehospitalizations Secondary: HF rehospitalizations, mortality, ER visits	No significant statistical differences
TELE-HF Chaudhry et al. (25)	Randomized, controlled trial	Telemonitoring of symptoms	1,653	Readmission + Death of any cause	No significant statistical differences
WISH Lynga et al. (26)	Randomized, controlled trial	Weight monitoring: automatically transmitted vs telephone messaging	344	Primary: Cardiac rehospitalization Secondary: Death from any cause, rehospitalization from any cause	No significant statistical differences
CHAMPION Abraham et al. (13)	Prospective, single blind trial, NYHA class III HF	Wireless monitoring of pulmonary artery pressures with CardioMEMS heart sensor	550	Rate of hospital admissions	Positive
TIM-HF Koehler et al. (27)	Randomized, controlled trial	Telemonitoring of weight, ECG, blood pressure	710	Death of any cause	No significant statistical differences
INH Angerman et al. (28)	Randomized, controlled trial	Telephone based monitoring of blood pressure, heart rate, symptoms Remote medication adjustments	715	Time to death or rehospitalizations	No significant statistical differences
CHAT Krum et al. (29)	Randomized, controlled trial	Telewatch system (symptoms assessment through questionnaires) + follow-up by nurses	405	Primary: Packer clinical composite score Secondary: rehospitalizations, deaths of any cause	No significant statistical differences
IN-TIME Hindricks et al. (30)	Randomized, controlled trial	Daily, multiparameter telemonitoring based on the ICD/CRT-D	664	Composite clinical score (including all-cause deaths and overnight hospital admission)	Positive
Blum et al. (31)	Randomized, controlled trial	Remote monitoring of symptoms, blood pressure, heart rate	204	Primary: Readmissions in the first month Secondary: all-cause hospitalisations, costs, mortality	Positive only for the primary end-point
EFFECT De Simone et al. (32)	Prospective, non-randomized	ICD telemonitoring	987	Mortality and rehospitalizations	Positive
OPTI-LINK HF Bohm et al. (33)	Randomized, controlled trial	ICD telemonitoring; Fluid status alert	1,002	Composite of deaths of any cause and cardiovascular rehospitalizations	No significant statistical differences
COMMIT-HF Kurek et al. (34)	Observational prospective cohort study	Daily ICD/CRT-D telemonitoring	822	Long term all-cause mortality	Positive
TIM-HF 2 Koehler et al. (35)	Randomized, controlled trial	Telemonitoring of symptoms, weight, blood pressure, heart rate	1,571	All-cause deaths or percentage of days lost due to unplanned cardiac readmissions	Positive
BeAT-HF Zile et al. (36)	Randomized, prospective, multicentre, controlled trial In patients with HFrEF	Baroreflex activation therapy with BAROSTIM NEO system (electrode placed on the carotid sinus connected to a subcutaneous pulse generator)	408	Changes from baseline in 6 months for quality of life score, 6-min walk test, NT-proBNP levels	Positive
Bowers et al. (37)	Randomized, prospective, controlled trial in patients with HFrEF	Active telemonitoring (symptoms, weight)	209	Primary: mortality rate Secondary: prescription of evidence based HF medication	No significant statistical differences
Ploux et al. (38)	Observational retrospective cohort study (one month before vs after the first French lockdown)	Multiparametric remote monitoring system (weight, blood pressure, heart rate, symptoms)	53	Medical contact index (cardiological/overall)	Decreased medical contact index after lockdown

**Table 3:** Summary of different hypertension telemedicine studies.

**Study**	**Study design**	**Assessed device/technique**	**Number of patients**	**End-points**	**Results**
Wakefield et al. (39)	Randomized, controlled trial−3 treatment groups (high intensity vs. low intensity *vs*. usual care)	Home telehealth device (daily BP monitoring and differentiated questionnaire low vs. high intensity group) and nurse management	302	Primary outcome: SBP change	Positive for the high intensity group
Hebert et al. (40)	Randomized, controlled trial−3 arms (nurse management vs. home monitoring *vs*. usual care)	Nurse counseling; Informing on strategies for controlling BP	416	Changes in SBP and DBP at 9 and 18 months	Positive for SBP in the nurse counseling group
Pan et al. (41)	Randomized, controlled trial−2 arms	Home telemonitoring for blood pressure (delivered by a GP, a hypertension specialist, a nurse)	198	Change in SBP at 1, 3, and 6 months	Positive
Margolis et al. (42)	Randomized, controlled trial−2 arms	Home BP monitoring and pharmacist management	450	Changes in SBP and DBP	Positive (for up to 24 months)
McManus et al. (43)	Randomized, controlled trial−3 arms (self-monitoring *vs*. telemonitoring *vs*. usual care)	Self-monitoring with electronic sphygmomanometer *vs*. telemonitoring via phone messages	1,003	Change in SBP at 12 months	Positive in both intervention groups
Mohsen et al. (44)	Randomized, controlled trial – 2 arms	Nurse counseling by follow-up phone calls	100	Changes in mean arterial pressure and body-mass index	Positive

**Table 4:** Summary of different atrial fibrillation telemedicine studies.

**Study**	**Study design**	**Assessed device/technique**	**Number of patients**	**End-points**	**Results**
CRYSTAL AF Sanna et al. (45)	Randomized, controlled trial−2 arms in patients with cryptogenic strokes (insertable cardiac monitor *vs*. conventional follow-up)	ECG monitoring with an insertable cardiac monitor	441	Primary: Time to first detection of AF in the first 6 months	8.9 *vs*. 1.4% (*p* < 0.001)
Stegmann et al. (46)	Randomized, controlled trial in patients with heart failure−2 arms (daily ECG *vs*. usual care)	Daily ECG transmitted to a telemedical center	879	Newly documented AF	Positive (AF three times more frequently detected)
Lambert et al. (47)	Unblinded, randomized, controlled trial in patients with recent AF ablation−2 arms (smartphone ECG *vs*. usual care)	ECG recordings once per week or during symptoms (Kardia Mobile devices)	100	AF detection during the 6-month follow-up period	No significant statistical differences
Fitbit Heart study Lubitz et al. (48)	Prospective single arm study	Fitbit trackers/ Smartwatches One week ECG patch monitor for patients with irregular heart rhythm detected by the device	1,057 eligible individuals	Positive predictive value (PPV) of the first irregular heart rhythm detection in atrial fibrillation detection	PPV 98.2% (95% CI: 95.5–99.5%)
Bernstein et al. (49)	Randomized, controlled trial−2 arms in patients with stroke due to large or small vessel disease (insertable cardiac monitor *vs*. conventional follow-up)	ECG monitoring with an insertable cardiac monitor	(455; 699 initially enrolled)	Incident AF (>30 s) in 12 months	12.1% *vs*. 1.8% (*p* < 0.001)
LOOP Diederichsen et al. (50)	Randomized, controlled trial, open label 1:3 (implantable loop recorder *vs*. usual care) in individuals with at least one risk factor for stroke	ECG monitoring via implantable loop recorder	493	Primary outcome: stroke or arterial embolism Secondary outcome: AF, mortality	Negative for the primary outcome 31.8% *vs*. 12.2% (*p* < 0.0001)–AF

Despite the fact that many studies failed to demonstrate a lack of positive effect on the primary end-point of all-cause mortality, several meta-analyses reported a reduction in mortality and heart failure-related hospital admissions when comparing telemonitoring with usual care (17). Although data regarding trends toward improved mortality are conflicting, these trials showed a significant reduction of HF hospitalization and reported improved quality of life in structural telephone support vs. usual care but did not improve the mortality trend (15, 51).

The results of these trials should be interpreted with caution as some of them are not sufficiently statistical powered, with different non-invasive monitoring techniques regarding symptoms and signs of HF (17). There is conflicting evidence of telemedicine benefits, mainly due to a lack of uniform methods, large heterogeneity of study design and different monitoring techniques among current studies (15).

The limitations of these studies due to the relatively small number of patients, different types of telemonitoring, and inadequate risk stratifications of the patients, lead to the difficulty to determine the extent of the beneficial outcome of the telemedicine (52). Many of these studies used non-invasive assessment of the relevant parameters to monitor: body weight, heart rate, heart rhythm, blood pressure.

Telemonitoring with implantable devices, either pacemaker (PM), implantable cardiac device (ICD), cardiac resynchronisation therapy (CRT), that have been placed for other indications, or implantable hemodynamic sensors that can measure intrapulmonary (CardioMEMS) or intracardiac pressures (V-LAP), lead to more timely recognition of serious arrhythmias or worsening HF (13, 16, 50, 53). The new implantable hemodynamic sensors detect the pressure minor changes and trend up to two weeks before the symptoms and signs like shortness of breath, congestion, and oedema emerge (16). However, there is a concern about the potential risk of overtreatment that may arise from the inappropriate use of remote monitoring strategies as the REM-HF study showed (15).

## Evolving Directions of Telemedicine

Similar to what happened more than 350 years ago, the COVID-19 outbreak is a turning point in the way we define and manage a new approach to cardiovascular patients. Many advanced technical and practical data regarding an approach toward remote and isolated patients have been gathered in the last 15–20 years. As always, the medical practice is on the frontline of change, but the professional decision is backed up by clear regulations from the regulatory and professional bodies of the National Societies of Cardiology (Italian, British, French, American, and Spanish), as well as the European Society of Cardiology (2, 16, 54, 55).

Telemedicine is based on video assessment, the electronic patient record (EPR) check, device/biosensors data and alerts. Tools previously used only by physicians are now useful in the hands of the local technician. In addition to electronic stethoscopes, it is our vision to use ultrasound technology, as well as the second generation of implantable biosensors.

One major direction of telemedicine development triggered by the COVID-19 outbreak is the online switch of the traditional cardiology face-to-face assessment (delivered directly to the patient as virtual video visit and teleconsultation), or by the intervention of a health professional (GP, ambulance crew or general medicine physician from small hospitals). A second expanding course of remote Cardiology consists of telemonitoring with new wearable devices, especially implantable biosensors assessing vital parameters and giving alerts for immediate management.

Telemonitoring of oxygen arterial saturation (SaO_2_) 24/7, in asymptomatic and mild SARS-CoV-2 cases isolated and confined at home, which generates alarms when values decrease and subsequently sent alerts to GP's surgery and community hospitals, allows immediate management measures, and transfer of the patients in specialized units, as the progression of COVID-19 is unpredictable.

A wireless electronic stethoscope or echocardiography transducer may assess the aortic, mitral and tricuspid valves. There are sensors and electronic stethoscopes, but large-scale self-check-up is still desirable. Currently, although not widespread, patients can place a digital stethoscope on the chest to allow the cardiologist to hear the heart sounds and murmurs in real-time on the other side of the virtual online. Digital stethoscopes that can simultaneously record ECG and phonocardiogram are already on the market. A real-time consultation and cardiac valve assessment, either with an electronic stethoscope or a wireless echocardiography transducer, will identify valve disease (aortic stenosis, mitral valve disease and changes in heart murmurs) of different causes (degenerative, endocarditis).

Chest pain and ECG patient self-assessment in acute chest pain will identify acute coronary syndromes (especially STEMI and NSTEMI). Initially, in 2005, telemedicine pre-hospital ECG shortened the delay to reperfusion and lowered mortality for STEMI (9).

The cardiovascular patients most affected by the outbreak are those with heart failure. The rates of hospitalization from heart failure are still high, with substantial financial costs. Heart failure remains a challenging scenario since it could overlap in clinical presentations with COVID-19 infection. On the other side, repeatedly admitted patients for acute decompensations are at risk of contracting the SARS-CoV-2 virus (56). The objectives of telemonitoring in heart failure are: prevention and treatment of disease exacerbations and promotion of patients' self-empowerment (15). Non-invasive telemedical systems have failed to prove reduced hospitalization rates in randomized clinical trials (57).

The situation changed when the first CardioMEMS heart sensor was implanted to monitor the pulmonary artery pressure among patients with a recent hospitalization for NYHA class III heart failure due to reduced ejection fraction. The implantable device provided daily pulmonary artery hemodynamic information allowing medication adjustment and a reduction in hospitalization for heart failure in randomized trials (13, 53).

**Figure 2:**
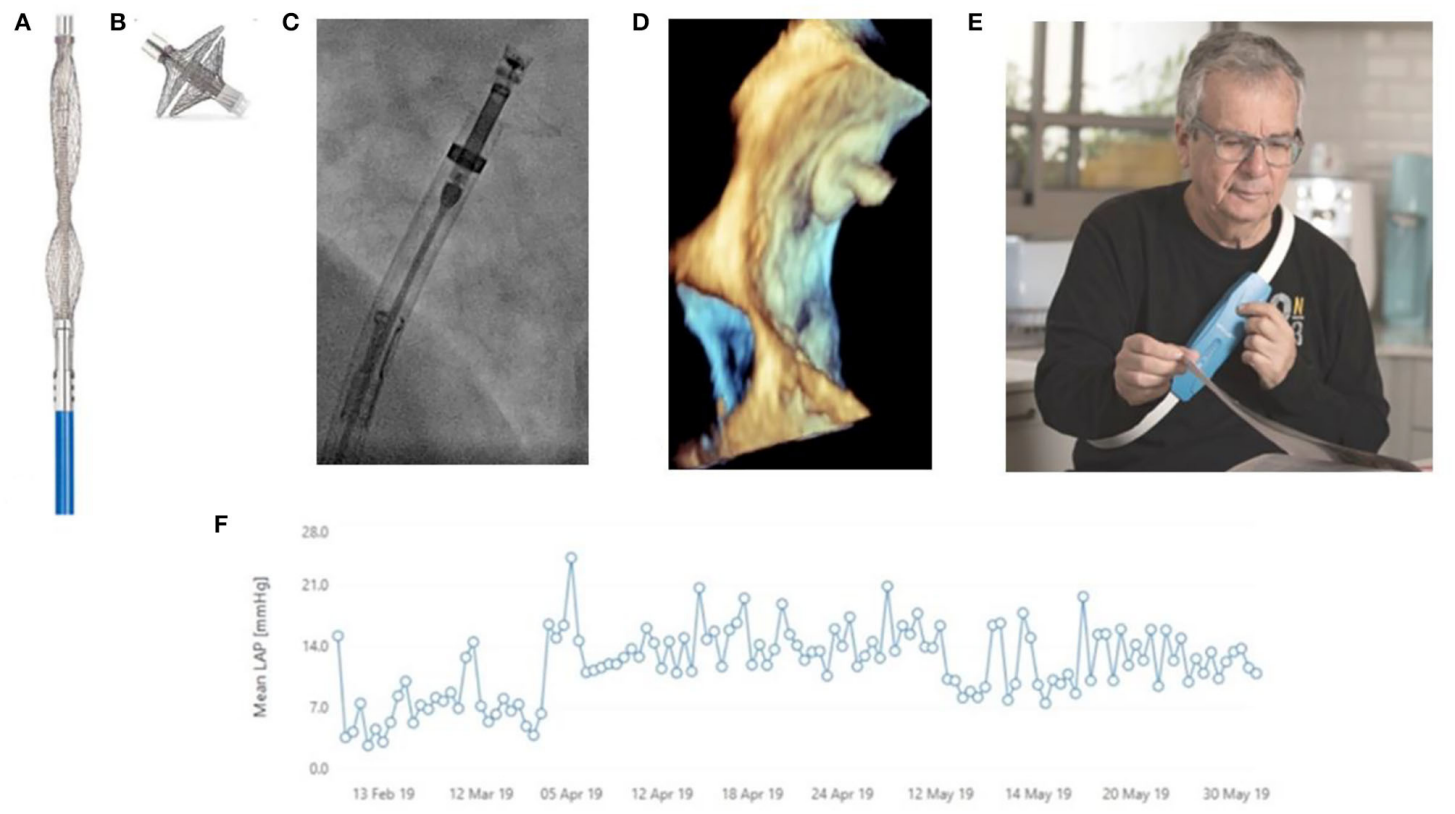
Vectorious Medical Technologies V-LAP™ wireless sensor, a second-generation heart failure (HF) monitoring system, used for remote LAP-guided Therapy Optimization during COVID-19 era at a patient with HF. **(A)** The unexpanded biosensor before implantation; **(B)** The biosensor after *ex vivo* expansion; **(C)** The catheter with the biosensor during deployment; **(D)** The 3D echocardiographic check of the biosensor positioning, as part of the interatrial septum (detail image) between the right atrium and left atrium (above); **(E)** The external unit (the belt for the patient) continuously captures and records the left atrial pressure; **(F)** The continuous diagram recording of the mean left arterial pressure monitored with V-LAP™. Alerts of the mean arterial pressure overpassing the normal values are transmitted via mobile to the cardiologist, allowing her/him to take action by optimizing the therapy. Reproduced from Di Mario et al. (58), with the courtesy of the authors.

Invasive haemodynamic monitoring with implantable hemodynamic monitors, like V-LAP in isolated cases, demonstrated the safety and feasibility of pressure-guided management in clinical trials. This showed a marked reduction in heart failure hospitalization (see Figure 2). These interventional implantable haemodynamic monitors provide proactive data on post-capillary left-sided filling pressures in a wireless, minimally invasive manner. This data is related to the pre-symptomatic hemodynamic changes and congestion, well in advance (with up to 21 days) of the clinical congestion symptoms (dyspnoea, body weight and heart rate change, peripheral oedema and jugular venous pressure raise), which present late in the course of decompensation (56–58). This would primarily be used to assess mean left atrial pressure, enabling the adjustment of heart failure medications on a daily basis to reduce the risk of an impending exacerbation and prevent medication overdose. However, in addition to that, the system is designed to detect and manage other common cardiac events.

There is still a lack of data regarding the studies that analyse the cost-effectiveness of different devices and assess the therapeutic management based on the telemedicine systems (17). Secure transmission and storage of medical data and information through text, regarding diagnosis, treatment and follow-up, are also important concerns of telemedicine (59).

Recent expert position papers consider the cost-effectiveness of telemedicine may be higher, and the proof of effectiveness might favor the development of reimbursement models, as new and more focused studies on the multimodal approach are using a closed-loop healthcare model called the Internet of Medical Things (60).

The application of new technologies does not only short-cut the delivery mechanisms of medical services to the patients, but transcends them at a superior level. We consider the cost-effectiveness ratio as the gold standard in assessing the expenditures, by maximizing the benefits of the patients with the least expensive technologies and procedures. Although telemedicine reduces the costs by decreasing the number of hospitalizations and visits to the physicians, overall, the costs of the care could increase due to the expenses of the new technologies, new devices, new interventional procedures, and data monitoring.

Recent data that prove the effect of technological advancements on financial issues in healthcare has exposed the divergence between the innovation cost and the change in medical resource utilization because of the clinical impact of this technology (61). We are rallying to the idea that more efforts should be made by the national public health authorities to better analyse the contribution of telemedicine and to find the best solutions for implementation (62).

Telemedicine needs validation through clinical trials for updating the recommendations and level of evidence in the clinical guidelines. Particular attention should be paid to patients with cardiovascular diseases and other debilitating chronic conditions like cancer, where telemedicine (including telemonitoring) can play a key role in the management of these patients (63). Due to its' ability to connect people and the rapidity of transmitting information, telemedicine comes forth as an important service for patients. But as any benefit comes with a cost, the long-term replacement of usual care with telemedicine where this is not entirely necessary, can lead to a sense of abandonment and isolation especially in the most fragile patients. It is important that clinician training highlights the limitations of telehealth and informs of alternative methods that can be used in this situation (64).

Access to healthcare has geographical, economic and time boundaries, and recently it has unveiled the additional challenge during the pandemic. Recent technological advances have made possible to provide healthcare to many patients who otherwise would not have been provided such an opportunity. As the emergence of mutant variants of SARS-CoV-2 and globalization increase the risk of new recurrent episodes or future pandemics, with close contacts often being quarantined at home as recently happened in the city of Shanghai, telemedicine is the bailout solution for many cardiovascular patients.

Besides its' role in lockdown, the need for telemedicine is a strong factor forcing the development of new guidelines on medical practice and documentation, reimbursement, privacy, and security of this service (64). Telemedicine emerged as a complementary service conceived to improve access and quality of care, reduce any inefficiencies in the healthcare system, that allow patients to better track their health and wellness at an affordable cost of healthcare (64).

Telemedicine started its' way to become a mainstream component of our health system, not only to deal with global and national emergencies like Covid-19 outbreak, but also as a part of normal healthcare strategy (65).

## Conclusions

Telemedicine is considered an important new player in the healthcare field. Its' slow recognition in diagnosis and management of cardiovascular conditions is in contrast with both the technological (including invasive telemonitoring) and commercial (plethora of new devices) progress made in the field.

Telemedicine is not just a conjectural approach related to the SARS-CoV-2 pandemic. Our perception is that the boundary between the pioneering of several cardiologists and the widespread application to the patient's bedside of innovative techniques is fading away, all these for the benefit of the patients. There is a telemedicine worldwide trend to encourage a switch from small and hesitating steps to a more consistent assessment of the patients, based on high technology and Interventional Cardiology.

Cardiovascular telemedicine, although having made significant progress in managing patients in lockdown, has many obstacles to overcome before meeting all the needs of the patients. The regulatory institutions could play an integrative role in leading the way for defining the framework of future telemedicine activities. However, to be successful, telemedicine needs a holistic approach, in which enlightened minds, with a clear vision, put together all the pieces, after having solved the ethical, legal, regulatory, technique and reimbursement issues.

The SARS-CoV-2 outbreak with all its tragedy served to reinforce the message that telemedicine services can be life-saving for cardiovascular patients. Once the Covid-19 era will melt down, telemedicine is likely to remain a complementary service of standard care.

## Author Contributions

L-NG, M-RC, and CDM designed the paper. L-NG, AB, IDK, and L-MM conducted the literature searches. L-NG, GS, MS, and CDM drafted the results. L-NG, L-MM, MS, and CDM contributed to parts of the discussion. L-NG, A-MS, L-MM, NF, M-RC, GS, AB, IDK, GGS, MS, and CDM provided critical analysis, revised the whole manuscript, and approved the final version of the publication. L-NG and CDM are responsible for all the revisions and remain in contact with the rest of the review team regarding status reports. All authors contributed to the article and approved the submitted version.

## Funding

This study was supported by Carol Davila University of Medicine and Pharmacy, Bucharest, Romania.

## Conflict of Interest

L-NG is a Consultant in Cardiology at the Elias University Hospital in Bucharest, and a Lecturer at the Carol Davila University of Medicine and Pharmacy, Bucharest, Romania. He also graduated International Economic Relations at the Academy of Economic Sciences in Bucharest with a degree in World Economy. He completed his training in Cardiology at hospitals in Birmingham and Oxford, and was subject of several honorary fellowships in Interventional Cardiology at Royal Brompton Hospital in London, UK, under the supervision of CDM. The remaining authors declare that the research was conducted in the absence of any commercial or financial relationships that could be construed as a potential conflict of interest.

## Publisher's Note

All claims expressed in this article are solely those of the authors and do not necessarily represent those of their affiliated organizations, or those of the publisher, the editors and the reviewers. Any product that may be evaluated in this article, or claim that may be made by its manufacturer, is not guaranteed or endorsed by the publisher.
